# Unhappy Patients Are Not Alike: Content Analysis of the Negative Comments from China's Good Doctor Website

**DOI:** 10.2196/jmir.8223

**Published:** 2018-01-25

**Authors:** Wei Zhang, Zhaohua Deng, Ziying Hong, Richard Evans, Jingdong Ma, Hui Zhang

**Affiliations:** ^1^ Institute of Smart Health School of Medicine and Health Management Huazhong University of Science and Technology Wuhan China; ^2^ Department of Business Information Management and Operations University of Westminster London United Kingdom; ^3^ School of Public Administration Guangzhou University Guangzhou China

**Keywords:** patient satisfaction, physician-patient relationship, Good Doctors website, patient complaint.

## Abstract

**Background:**

With the rise in popularity of Web 2.0 technologies, the sharing of patient experiences about physicians on online forums and medical websites has become a common practice. However, negative comments posted by patients are considered to be more influential by other patients and physicians than those that are satisfactory.

**Objective:**

The aim of this study was to analyze negative comments posted online about physicians and to identify possible solutions to improve patient satisfaction, as well as their relationship with physicians.

**Methods:**

A Java-based program was developed to collect patient comments on the Good Doctor website, one of the most popular online health communities in China. A total of 3012 negative comments concerning 1029 physicians (mean 2.93 [SD 4.14]) from 5 highly ranked hospitals in Beijing were extracted for content analysis. An initial coding framework was constructed with 2 research assistants involved in the codification.

**Results:**

Analysis, based on the collected 3012 negative comments, revealed that unhappy patients are not alike and that their complaints cover a wide range of issues experienced throughout the whole process of medical consultation. Among them, physicians in Obstetrics and Gynecology (606/3012, 20.12%; *P*=.001) and Internal Medicine (487/3012, 16.17%; *P*=.80) received the most negative comments. For negative comments per physician, Dermatology and Sexually Transmitted Diseases (mean 5.72, *P*<.001) and Andrology (mean 5, *P*=.02) ranked the highest. Complaints relating to insufficient medical consultation duration (577/3012, 19.16%), physician impatience (527/3012, 17.50%), and perceived poor therapeutic effect (370/3012, 12.28%) received the highest number of negative comments. Specific groups of people, such as those accompanying older patients or children, traveling patients, or very important person registrants, were shown to demonstrate little tolerance for poor medical service.

**Conclusions:**

Analysis of online patient complaints provides an innovative approach to understand factors associated with patient dissatisfaction. The outcomes of this study could be of benefit to hospitals or physicians seeking to improve their delivery of patient-centered services. Patients are expected to be more understanding of overloaded physicians’ workloads, which are impacted by China’s stretched medical resources, as efforts are made to build more harmonious physician-patient relationships.

## Introduction

### Benefit of Web-Based Patient Complaints

The identification and recording of patient complaints is vital for improving the quality of health care services and maintaining good physician-patient relationships [1]. However, not all patients complain when they are dissatisfied. Previous studies [2,3] on quality management have revealed that only one-third of patients complain when they experience unsatisfactory service. Reasons for patients not complaining include lack of contact information for customer complaints offices, complicated complaint procedures, and insufficient transparency in dealing with complaints [4-6]. With the growth in worldwide Internet availability and usage, a behavioral shift is identified where people are moving from traditional offline complaint channels to expressing their views in relation to unsatisfactory health care experiences via the Internet. Such channels provide an alternative approach for patients to discuss their substandard medical experiences and share them with others, especially when they feel the service provider failed to take effective action [7]. Compared with on-site complaints, patients benefit from increased time and description allowance when complaining online. Salma et al [8] conducted a qualitative study into patients’ attitudes toward submitting Web-based feedback and ratings to general practitioners (GPs) in England; they suggested that patients leave comments online mainly for one of the following three reasons: (1) the ability and ease of giving it remotely, (2) availability to the public, and (3) the perceived serious attitude of the GPs toward the Web-based comments.

### Web-Based Patient Comments and Their Impact

Undoubtedly, comments posted online by dissatisfied patients exert a potentially greater impact on reviewer behaviors relevant to medical scenarios. Most of the time, these comments could be identified in specific physician rating websites (PRWs) where patients can share their medical experiences with a certain physician, serving as a reference for medical decision making [9]. PRWs enable patients to post comments relating to their experiences and emotions following appointments online [10], which identifies patients’ actual experiences of health care providers. With this type of information sharing, not only can physicians be better informed about patient concerns [11], but they can also benefit from being able to offer the most appropriate physicians and facilitating the communication between them. Previous research [12] has concluded that comments for physicians are positive in nature. A content analysis of 3000 narrative comments posted on a German PRW revealed that the majority of comments were positive (2400/3000, 80%), with 16% (480/3000) being negative and 4% (120/3000) neutral [13]. Similar results were found on the largest doctor review website in China, the Good Doctor [14]. Other researchers have further explored PRWs via new analytical techniques [15-20]. Greaves et al [16] adopted a machine-learning approach to classify 6421 comments obtained from the English National Health Service website, splitting them into positive and negative posts. Another study [18] revealed that complaints relating to access to appointments, appointment waiting times, and time spent with a physician were viewed as most important. Li et al [21] examined the proportion and position of Web-based negative reviews and their effect on patient decision making. They found an increase in the number of negative reviews and identified that the higher the position of a negative review, the greater was the reluctance of patients to use a physician’s services. It is evident that patients favor physicians who receive the most positive comments over those with negative or dissatisfied comments. However, negative or dissatisfied comments can hardly be avoided, considering the unpredictable behavior of individual patients following their health care experience. Thus, readers of Web-based reviews must be cautious when encountering negative or dissatisfied comments when making their judgments.

Negative comments posted may harm or create a damaging image of physicians and increase the dissatisfaction of patients toward the physician or hospital [22]. A study of GPs in England suggests that Web-based negative comments may affect a GP’s confidence and self-esteem and lead to self-defense during their future work [23]. Extant studies [9,10,12,14,18] have predominantly focused on large-scale investigations of PRWs and the overall attitudes of comment providers, with little attention being given to the detail of negative comments posted. This study aims to analyze factors associated with negative comments posted online and provide empirical evidence for the understanding of unhappy patients and their comments. The research questions posed in this investigation include the following:

RQ1: What do patients complain about online in relation to their physicians?

RQ2: How can we improve the physician-patient relationship through the lens of Web-based negative comments?

## Methods

### Data Collection

**Figure 1:**
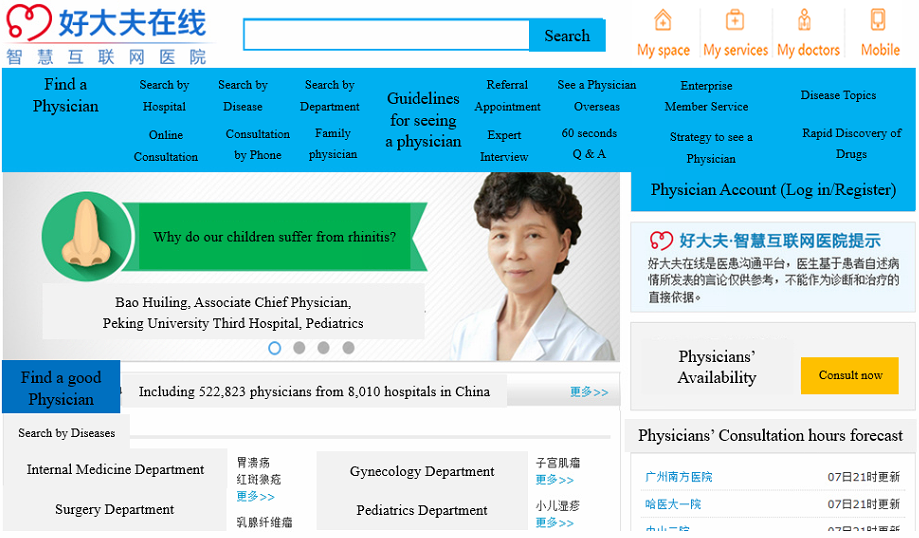
The home page of the Good Doctor website (accessed September 8, 2017).

Data were collected from the Good Doctor website, one of China’s largest online physician-patient communication platforms (see Figure 1 for home page visual). The platform was established in 2006 and currently has 7794 hospitals and over 500,000 physicians registered to the service [24]. The website enables patients to access three types of services: (1) health information search, (2) medical consultation, and (3) patient feedback. For the health information search, users are able to obtain medical knowledge, medical news, and expert opinions. For the medical consultation service, patients can consult specific physicians via picture, telephone, or videoconference. In addition, online booking of appointments for referrals, delivery of drugs, arrangement of remote medical consultations, and the issuing of electronic prescriptions are also possible. For patient feedback, patients can rate their physicians and submit comments. As such, Good Doctor website is the first nationwide online platform for patients to share their experiences with physicians in China. Feedback mechanisms allow patients to vote, comment, write thank-you letters, and send electronic gifts to their physicians. The website’s rating system automatically recommends good doctors based on patient ratings and provides a comprehensive evaluation of the expertise of physicians, as well as a hospital’s reputation.

The Good Doctor website declares that all personal patient information contained on their website is removed automatically, including medical profiles and consultation records. All the content generated by the website’s users and doctors is regarded as shared property between its users, doctors, and the website. In addition, content is made publicly available for the common good, but commercial use is prohibited.

Given that China’s medical resources are unevenly distributed, economic prosperity and administrative powers always imply that abundant medical services are available. In this study, we focus solely on Beijing, China’s capital, where the leading and preeminent hospitals in China are situated. As a pilot study, comments were collected on physicians from 5 reputable tertiary referral hospitals in Beijing, including China-Japan Friendship Hospital, Chinese PLA General Hospital, Peking University First hospital, Peking University Third Hospital, and Peking Union Medical College Hospital. A predesigned Java-based Web crawler was utilized to obtain all webpages concerning the 5 hospitals on Good Doctor website in September 2016. Data collected were stored in a MySQL database through a process of page parsing and information extraction. Data consisted of 140,591 patient comments relating to 5727 physicians posted on the physicians’ home page.

On the Good Doctor website, patients can rate their satisfaction with medical services provided on the following scale: very satisfied, satisfied, ordinary, no comment, and dissatisfied; this rating relates to the physician’s attitude and therapeutic effect in his or her latest consultation. As our research concerns patients’ dissatisfaction, we have selected patient comments where at least one item (physician attitude or therapeutic effect) was considered to be unsatisfactory. After filtering “noisy” and null data by text preprocessing, we obtained 3012 dissatisfied comments (see Table 1 for details). Among them, 1565 (51.96%, 1565/3012) patients rated their experience as unsatisfactory in relation to the physician’s attitude and manner. For the remaining comments, 67 out of 1447 (4.63%) indicated that they were either very satisfied or satisfied with the physician’s attitude; in terms of therapeutic effect, the figure was 197 (12.59%, 197/1565), which is much higher than that recorded for “physician’s attitude.” We compared the two groups of negative comments toward physician attitude and manner, and no statistical difference was found (*P*=.90). All comments concerned 1029 physicians in total (mean 2.93 [SD 4.14]). The average length of a comment is 195.83 words (min=13, max=3345, [SD 189.19]). For each physician, the number of negative comments they received ranged from 1 to 69. After grouping patients using the classification of the Good Doctor website, we found that the Department of Obstetrics and Gynecology received the most negative comments with 606 (*P*=.001), followed by the Department of Internal Medicine with 487 (*P*=.08). Meanwhile, for the number of negative comments per physician, the Department of Dermatology and Sexually Transmitted Diseases (STD) and Andrology ranked the highest with 5.72 and 5 comments per physician, respectively. Table 2 provides further details.

**Table 1:** Overview of the data.

Dissatisfied items	Very satisfied	Satisfied	Ordinary	No comment	Dissatisfied	*F* value	Significance
Attitude	28	39	224	579	1565	0.02	.90
Effect	63	134	355	25	1565		

**Table 2:** Number of negative comments by departments.

Department	Number of negative comments	Number of physicians	Negative comments per physician	Standard deviation	*P* value
Internal Medicine	487	204	2.39	3.11	.08
Surgical Department	356	164	2.17	1.99	.02
Obstetrics and Gynecology	606	132	4.59	7.24	.001
Reproductive Center	103	23	4.48	5.25	.08
Pediatrics	104	45	2.31	2.00	.32
Orthopedics Surgery	162	64	2.53	2.83	.45
Ophthalmology	68	42	1.62	0.99	.04
Stomatology	75	39	1.92	1.86	.13
Otorhinolaryngology (ear, nose, and throat) and Head and Neck	128	41	3.12	2.52	.77
Oncology	28	11	2.55	1.75	.76
Dermatology and Sexually Transmitted Diseases	372	65	5.72	6.76	<.001
Andrology	125	25	5.00	6.73	.02
Psychiatry	10	5	2.00	1.41	.62
Traditional Chinese Medicine	90	38	2.37	1.52	.41
Integrated Traditional Chinese Medicine and Western Medicine	4	4	1.00	0.00	.35
Intervention Therapy	8	4	2.00	2.00	.65
Rehabilitative Medicine	10	3	3.33	4.04	.87
Sports Medicine	31	19	1.63	0.76	.17
Anesthesiology	19	9	2.11	3.33	.56
Occupational Diseases	1	1	1	—	—
Medical Imaging	14	10	1.40	1.27	.24
Others	211	81	2.60	2.41	.48

### Data Analysis

**Figure 2:**
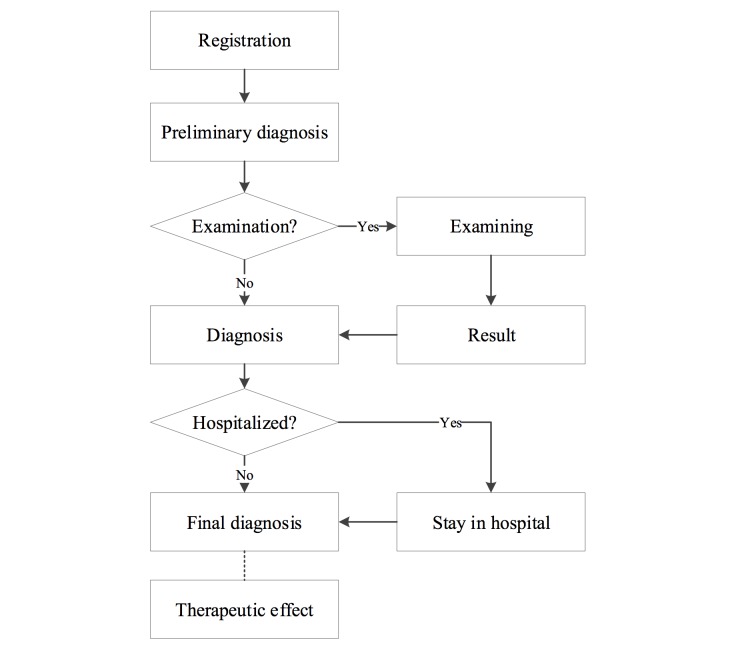
Workflow of medical consultation in a typical tertiary referral hospital.

Content analysis was used to explore the factors associated with patient dissatisfaction [25,26]. Reader et al [27] developed a coding taxonomy for patient complaints through a systematic review of 59 studies. Following thematic analysis and grouping, they conceptualized three distinct domains of complaint: (1) safety and quality of the clinical care received, (2) the management of health care organizations, and (3) problems associated with health care staff-patient relationships. On the basis of their framework and the workflow of medical consultation in China (see Figure 2), we developed a coding framework with five dimensions of patient dissatisfaction that occurred before, during, or after the patient received health care from a specific physician, consisting of (1) the physician’s attitude, (2) therapeutic effect, (3) ignorance of patient, (4) limited treatment time, and (5) misconduct or bad attitude of the nurse and/or other staff.

**Figure 3:**
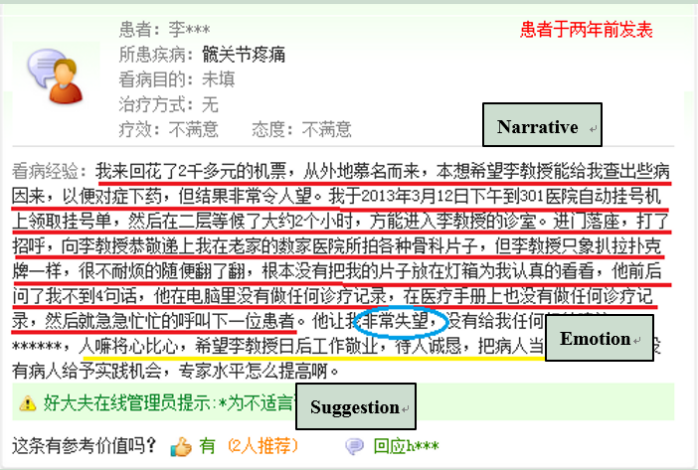
A sample of negative comments from the Good Doctor website.

Patients and/or their caregivers submit comments online to advise those seeking health care from doctors when experiencing similar diseases or symptoms. Generally, negative comments can be divided into three types: (1) content, including an explanation of their medical experience; (2) emotional complaints relating to the service received; and (3) suggestions for health care delivery improvement. A sample of negative comments is provided in Figure 3, whereas Table 3 provides the sample in English, accordingly. Narratives describe the medical experience honestly, whereas emotional words are highlighted in the emotion type. For suggestions, the commenter suggests possible solutions for health care delivery improvements. These three types may not always be identifiable in each comment, whereas sometimes, commenters could mention all of them. Two research assistants (RAs), both with a medical informatics background, were involved in the coding process, following a training session. They independently coded a random selection of 9.99% (301/3012) of the total comments within the pilot framework. If concepts were beyond the previous coding scheme, the two RAs discussed adjustment until consensus was reached. Any discrepancy or disagreement was solved by WZ, the lead author of this study. After the independent coding of the 301 comments, the final coding framework was formed, and the intercoder reliability was shown as Cronbach alpha=.82, indicating high credibility. Finally, one RA coded the remaining comments using the newly developed framework; the coding framework is presented in Table 4.

### Coding Framework

#### Identity

The identity of the commenters is defined by the relationship between the commenter and patients. The identity of commenters was coded into four types according to their closeness to the patient: (1) commenters as patients themselves; (2) commenters as a patient’s close relative, including spouse, parent, or a grown-up child; (3) commenters as a patient’s other relative; and (4) for friends and other relationships not specified. Meanwhile, some comment providers also identified themselves as “traveled patients,” visiting hospitals in Beijing because of their reputation in specific medical fields. Therefore, we coded (1) for traveled patients and (0) for local patients.

#### Dissatisfaction

For the coding scheme relating to dissatisfaction, we followed the workflow of a health service delivery provider in a Chinese tertiary referral hospital. Generally, if a patient wants to see a doctor, he or she must register for that doctor’s surgery (doctor’s office) before a face-to-face consultation can occur. We coded this as the “premedical consultation” stage. During this stage, complaints include topics such as registration and waiting room issues and time taken before consultation. During medical consultation, four substages are identified, ranging from (1) overall perception, (2) preliminary diagnosis, (3) examination, to (4) the closure of consultation. Overall perception refers to the immediate evaluation of a physician’s attitude and their communication with the patient. Preliminary diagnosis relates to the first contact experienced between the physician and patient, where the physician commonly employs four techniques of diagnosis, that is, to look, listen, question, and feel the pulse of the patient, to obtain an individual’s information. In the examination stage, a medical device is applied to the patient, such as computed tomography film or a blood pressure monitor. The closure of consultation refers to when the patient is about to leave the hospital, with complaints typically concerning bills. After the consultation, patients may start to evaluate the effect of the treatment, if any. We coded this as “post consultation,” with a focus on the patient’s perception of effect. Each of the complaint areas is coded with 4 to 9 items.

**Table 3:** Three types of negative comments.

Types	Definition	Example
Narrative	Commenter describes their medical experience in an objective manner.	*I waited 2 hours to see Dr Li, but he just flipped through my results like he was playing poker for a few seconds and simply called the next patient after me in less than four sentences with me. He didn’t amend any of my medical records.*
Emotion	Commenter uses emotional words to express their dissatisfaction.	*I was very disappointed and he failed to give me any good advice.*
Suggestions	Commenter provides suggestions in the hope of health care delivery improvement.	*Dr Li should learn to feel for others and work harder in the future. I hope he could be more kind to patients next time.*

**Table 4:** Coding framework for negative comments.

Stages	Complaint areas		Code
Premedical consultation	Registration		1=hard to register, 2=long waiting hours, 3=high cost, 4=others
	Waiting for consultation		1=long waiting, 2= chaotic queuing
Medical consultation	**Overall perception**		
		Physician’s attitude	1=impatience, 2=disrespect patients, 3=not caring patients, 4= unavailability of physicians on duty, 5=do irrelevant things, 6=others
		Patient-physician communication	1=lacking communication, 2=not answering, 3=no time for communication, 4=others
	Preliminary diagnosis		1=ignorance of medical records and previous reports, 2=no observation (dermatology issues), 3=no inquiries, 4=others
	Examinations		1=lacking basic examinations, 2=too many examinations, 3=rude examinations, 4=repeated and inappropriate examinations, 5=long wait hours for the results, 6=no analysis for the results, 7=high cost. 8=privacy issue, 9=others
	Closure of consultation		1=no lifestyle advice, 2=no analysis before medical advice, 3=no diagnosis conclusion, 4=high cost of medical, 5=short time for diagnosis, 6=no treatment plan, 7=misdiagnosis
Post consultation	Patient’s perception of effect		1=no effect or little effect, 2=worse than before, 3=inappropriate treatment plan, 4=others

## Results

### The Majority of Commenters Were Patients Themselves, With Several Comments Being Contributed by Accompanying Persons During Medical Consultation

**Figure 4:**
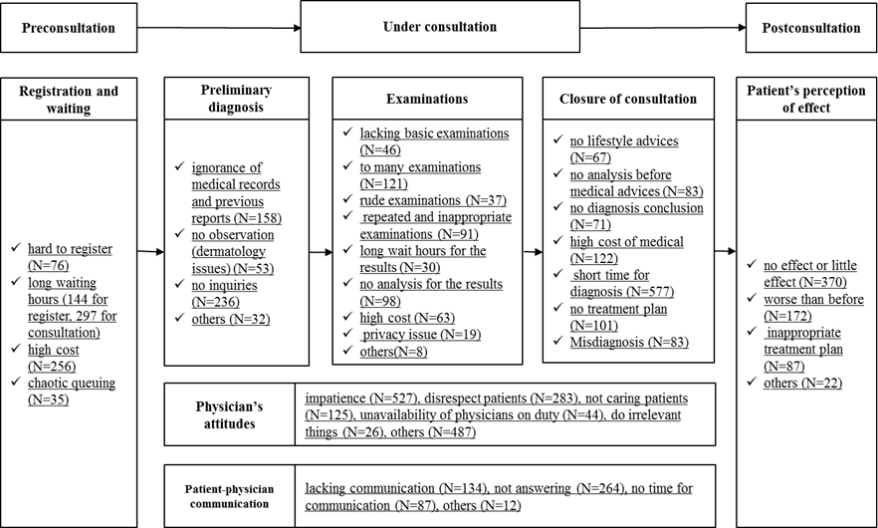
Main results of the coding.

A total of 86.22% (2597/3012) of commenters shared their experience as patients, contributing direct feedback relating to the perceived effectiveness and efficiency of the medical service they received (see Figure 4 for details). For the accompanying persons, the largest group were the patient’s grown-up children (227/3012, 7.54%), followed by the patient’s parents (149/3012, 4.95%). With the aging population of China, the country has a high percentage of “older” inhabitants, with the chance of this age group contracting diseases, particularly chronic illnesses, being considerably higher than their younger counterparts. It is common for young adults in China to accompany their older parents when visiting a doctor. Meanwhile, taking care of a child’s health is also an important responsibility in Chinese family culture, and parents typically accompany their children during hospital visits and treatment.

In addition, 228 (7.57%, 228/3012) dissatisfied commenters were “traveled patients.” This group had traveled to one of the five highly rated hospitals in Beijing because of the excellent level of medical services perceived to be provided there. These patients are more concerned with the quality of medical consultation received because of the additional costs incurred from the long distance traveled and accommodation required.

### Many Complaints Posted Related to Long Waiting Times Experienced and High Costs Associated to Registration During the Premedical Consultation Stage

Of all negative comments mentioned during the premedical consultation stage, long waiting times accounted for the majority of complaints, representing 7.9% (350/441) of total issues. It is common for patients to feel upset and/or anxious when suffering from a poor health condition that would cause them to have little tolerance for a long waiting time. High costs relating to registration fees is second, whereas 29 comments highlighted a special group—ticket touts. Ticket touts, or *Huangniu* in Chinese, in the context of hospital settings, refer to an individual or group of people who work as agents for patients to acquire their desired, but usually hard to obtain, reservation tickets to see renowned doctors. If ticket touts are able to obtain the required tickets, they may charge the patients much more than the normal ticket price. For example, a reservation ticket costs between 7 to 14 yuan but could sell for at least several hundred yuan and sometimes thousands. On the Good Doctor website, patients expressed their dissatisfaction with this unethical practice. They believed that ticket touts have disturbed the regular pricing strategy and cause increased difficulties during registration. Hard to register (N=76) was also specified. Cutting in lines or disorderly queuing systems (N=35) may further worsen the situation. One grown-up child accompanying their parent noted the following:

We have tried quite a lot to reserve Doctor X. We waited for a whole night to get registered...I do not know what happened to the auto-calling queuing system and we waited until 11 in the morning though there were still quite a few patients ahead of us.

### Physician Attitudes Toward Patients Are of Great Concern, With Impatience and Disrespect Being Identified Most Often

The attitude of physicians toward patients in medical settings can be considered crucial, with a total of 1492 complaints (26.91%, 1492/5544) identified. Among them, the impatience of physicians was mentioned most frequently (527/5544, 9.51%), with impatience relating to physician responses to patient questions, explanations of medical results and daily communication with patients being identified. Impatience was perceived by commenters as emotional abuse and could exert substantial influence on a patient’s mood. Although the comments mainly concerned the attitude of physicians, 17 commenters also blamed the physician’s assistants or medical staff for poor attitude. The disrespecting of patients came second (283/5544, 5.10%); disrespectful behaviors are distinguished from general bad attitudes, which encompass detesting, criticizing, and/or blaming of patients, with or without verbal abuse. Some “traveled patients” complained that their physicians demonstrated regional discrimination and perceived unfair treatment compared with local inhabitants of Beijing. In addition, another 125 (4.15%, 125/3012) comments emphasized little care perceived from physicians. Patients can be considered emotionally fragile and sensitive and may need more attention than what has previously been experienced. One patient commented the following:

We went to see Doctor Sun in the anus-intestines Department...he just asked us a few questions with a bad attitude. We felt awful because of his impatience...We hope that Dr Sun could think in the patients’ shoes and have a parental heart.

### Poor Physician-Patient Communication Was Highlighted by Patients and Their Accompanying Persons

Due to the knowledge gap between physicians and patients, physicians typically dominate the conversations and may not involve patients in decision making. A total of 264 (8.76%, 264/3012) commenters posted negative comments based on the physician not answering their questions, whereas 87 commenters felt that they experienced no communication between themselves and the physician. In addition, another 134 (4.45%, 134/3012) posted complaints about insufficient time spent communicating. One commenter wrote about their regret when their mother was diagnosed with synovitis, as follows:

Five months after the diagnosis...we waited for a long time and managed to see the doctor around 5 PM. We wanted to talk with the doctor, but it seemed that he did not want to talk with us at all. He did not want to answer our questions and demanded us to pack the film. He interrupted our several attempts to ask questions and we had to give up...We felt helpless as a patient.

### No Inquiry From Physicians Caused Serious Dissatisfaction During Preliminary Diagnosis

The preliminary diagnosis stage is the first form of contact experienced between the patient and physician before a further examination with medical devices is conducted. At this stage, physicians usually perform a series of diagnostic tasks such as looking, smelling, questioning, and taking the pulse of the patient to find out the cause, severity, or development of the disease or symptoms. A total of 236 (7.84%, 236/3012) commenters posted that their physician had not inquired about their disease or symptoms, whereas another 158 (5.25%, 158/3012) mentioned that no concern for medical records or previous reports was shown. These behaviors could lead to an unprofessional image of the health care providers. Patients may also feel that physicians are too busy to draw an informed conclusion without collecting physical evidence, which may lead to a reluctance to follow physician advice. One patient noted the following:

My son has had neck ache for years. I came for his reputation in pediatrics. However, he just glimpsed at the CT film for a second and did not inquire about my kid’s condition at all...I felt that he never cared and just concluded that my kid was OK. That’s really unacceptable.

### Too Many Examinations Conducted and No Analysis of Results Were Major Factors Associated With Dissatisfaction During the Examination Stage

A total of 121 (4.02%, 121/3012) commenters stated that the number of examinations conducted on them were too many, whereas 91 patients thought that their examinations were repeats of previous examinations or unnecessary. Increased examinations mean high costs for patients, and 63 commenters believed that the chaotic arrangement of examinations resulted in them paying more money. In addition, 98 commenters expressed unhappiness because of their physicians not analyzing their results. A total of 30 commenters felt that the waiting time for their reports was unacceptable. Regarding privacy issues experienced during examinations, gynecology patients constantly mentioned the problem of more than one patient being jointly examined at the same time. Rude behavior such as too much strength or pressure used during examination was also reported in 37 cases. One commenter noted the following:

I went to the doctor last year. He asked me to go through a series of checks. He instructed me to take CT films and B-ultrasound each, twice. Others, like gastroscopy...the staff examined me as if I were an object...It was a hurtful experience in that hospital.

### Limited Time Spent in Medical Consultation Was the Main Complaint Reported on at the End of the Medical Service

A total of 577 (19.16%, 577/3012) commenters discussed the duration of medical consultation, which may explain the perceived “rushed” attitude of physicians and the insufficient communication experienced. Further exploration reveals that patients are more likely to post negative comments if long waiting times and short medical consultation time are experienced together. One commenter noted the following:

My father has insomnia...and I decided to take him to see this doctor. We have waited for 3 hours, but the whole medical session did not last longer than 8 minutes. I felt so upset.

Furthermore, 122 (4.05%, 122/3012) patients complained about costs associated to medical fees. Another 101 (3.35%, 101/3012) commenters were disappointed because of no advice being offered during treatment, whereas 83 patients found it hard to accept their medical schedule without any medical analysis being explained to them. A total of 83 patients were dissatisfied because of not receiving a conclusive diagnosis by the end of their consultation.

### Therapeutic Effect Was Considered the Most Important Experience During Postmedical Consultation

Following consultation, patients cared most about the improvement in their health condition. A total of 370 (12.28%, 370/3012) patients rated therapeutic effect as unsatisfactory because of the results they expected post consultation. A total of 172 (5.71%, 172/3012) commenters mentioned that their health condition had become worse since their premedical consultation. In addition, 87 patients believed that the medical schedule advised may be inappropriate. Some claimed that after consulting another physician, who made a different diagnosis, their disease was eventually cured. It is highly possible that patients feel unstable and give a low rating to physicians if they perceive little therapeutic effect. Sometimes, therapeutic effect may outweigh attitude problems experienced. For example, one patient mentioned the following:

Dr Song is very careful and has a perfect attitude toward us, but he cannot solve our problem. A simple allergic rhinitis, he fails to diagnose, let alone the other ones. He just gave me some vitamins and sent me home.

## Discussion

### Dissatisfied or Unhappy Patients Are Not Always Alike, With Reasons for Feeling Dissatisfied Being Individual to Each Patient

Patient dissatisfaction is experienced throughout all stages of medical consultation. Although efforts have been made to classify all negative factors that appeared in the 3012 comments, it is still extremely difficult to present them with limited codes. Many complaints are intertwined; for example, perceived high costs can be associated with either registration, at the examination stage, or as part of the closure of consultation. If patients perceive that the medical consultation was expensive, they are more likely to complain about the short duration of consultation, the physician’s bad attitude, and/or poor therapeutic effect. However, for reputable tertiary referral hospitals in Beijing, a comparatively higher cost should be expected compared with ordinary hospitals; this is because of patients having higher expectations when they visit physicians in reputed hospitals, as they hope doctors can solve their problems that have not been addressed at other less reputable hospitals. With high expectations, several groups of patients are seen to be difficult to satisfy, including patients with chronic or complicated diseases, “traveled patients,” and very important person registrants. Meanwhile, accompanying persons who have strong feelings for their beloved ones, usually old parents or children, are easily irritated [28]. For both physicians and the hospitals, special attention is required for these groups of patients to avoid possible physician-patient conflicts [29].

Interestingly, we found that the Departments of Obstetrics and Gynecology and Internal Medicine received the most negative comments. This may be because of physicians in these departments receiving the highest number of comments in general on Good Doctor website [14,19]. In addition, the department staff also imply that their patients may be hard to please. For the former, perinatal care usually involves more family members, and their expectancy of a new baby makes them more sensitive to the medical care provided, especially for the primipara. For the latter, as Internal Medicine is generally difficult to observe, compared with Surgeries, patients may be more critical and easily irritated; this could also explain why departments such as Dermatology and STD and Andrology were the most complained about department per physician. Specifically, dermatology and plastic surgery correlate with each other, and patients may require an increased time to become accustomed to physical and mental changes; for STD or Andrology, no immediate cures are available, and patients have a strong concern about their privacy.

### Patient Complaints Are Complicated and Contradictory

With the diversity of patient complaints recorded, it is evident that patient perceptions are often different. For example, for the same physician, one patient may perceive none or few examinations as a “rushed” service to make decisions and move on to the next patient, whereas others may loathe excessive or inappropriate examinations. An example of this is *Jiahao* (adding reexamination patients), where physicians will add patients for reexamination to the top of their patient list, saving time for the patient. However, some patients regard *Jiahao* as unfair and perceive that it could result in much longer waiting times than usual. Since the duration of medical consultation for a physician is a fixed period every day, if the physician sees one more patient, it means less time for another patient. In this regard, it brings us to the issue of “justice,” in the context of medical services. Each patient may wish greater attention is given to them by their physician, so it is often difficult for them to tolerate other patients who take up a physician’s time. If this happens, the patient may perceive that they have been unfairly treated and blame the physician because of them having the power to manage the time of each appointment. However, it is unrealistic to implement a rule that the physician must spend a certain amount of time with a patient because of patient diversity, such as severity and types of diseases. In general, each patient’s preferences and understanding of a medical consultation is different, and no simple explanation for each patient’s dissatisfaction can be identified. Furthermore, although patients tend to avoid physicians with negative comments, it is sometimes unavoidable because of various reasons. One comment revealed the following:

I searched almost all the doctors who might solve my problems online and found everyone said X was not a good one. I wish I could avoid her, however the only one I could see is her upon my arrival.

### Incorrect Medical Advice, Overcrowded Medical Resources, and Policy Failures in Beijing Hospitals Jointly Contribute to the Dissatisfaction of Patients

For most Chinese people, modern medical treatment is perceived to be lifesaving, using the technologies or skills possessed by physicians. Many Chinese people believe that hospitals and their physicians should solve all patient problems; if not, they perceive the hospital or physician is not good enough. With this in mind, patients believe that the best, most highly rated hospitals or physicians are rare, and their health conditions may be best addressed, as long as they are willing to pay more. In the case of tertiary referral hospitals in Beijing, people are convinced that they are the best hospitals in China and wish that they can go to the best hospitals whenever they are sick. As a result, patients expect hospitals to be overcrowded, with increased waiting times and physicians spending less time with each patient. However, modern medical treatment does not provide a cure-all approach. A patient with the common flu will take approximately 1 week to recover. Seeing the best doctor in the best hospital will not reduce this time and simply deprives those who require medical assistance most of the medical resources. In recent years, China has started to implement a hierarchical medical scheme; this aims to divert patients in tertiary referral hospitals to primary hospitals and community hospitals. However, this policy is still in its infancy. Most people distrust physicians in primary or community hospitals and feel reluctant to see a doctor in these hospitals [30]; this causes an unexpected influx of patients at tertiary referral hospitals, which must satisfy emerging health demands from the public. With the increasing prosperity of China, Chinese people are now more concerned about their health conditions and have more demands or expectancy toward physicians that is often very difficult to satisfy.

### Blame the Physicians, but Also Try to Sympathize With Them

Throughout the medical consultation, physician-patient communication is still a key factor that affects patient satisfaction. Patients are human beings, and their perception of medical consultation is mainly built upon the communication had with their physicians. For the physicians, a warm heart and a good, friendly attitude is very necessary [31]. In China, hostility between physicians and patients is largely caused by the limited medical resources available to the physician [32]. These limited resources cannot satisfy the emerging demands for health care, with medical agents such as ticket touts causing extra strain on the delivery of medical services. In the case of ticket touts, they usually sell their “tickets” through one of four approaches: (1) offline queuing. Ticket touts are familiar with the rules of ticket delivery and arrive early to ensure the first position in the ticket queue. Their appointment ticket can then be sold to any paying patient, (2) tickets from internal resources. Ticket touts take full advantage of their *guanxi* or good relationship with physicians, nurses, and internal staff to obtain internal or additional tickets. It is sometimes possible that internal staff may collaborate with ticket touts, (3) ticket-buying plug-ins or mobile apps. As an increasing number of hospitals have embraced the Internet for ticket delivery, ticket touts provide paid services for making appointments for patients online, and (4) stocking up and reselling tickets. For this approach, the ticket touts need to find bugs or “loopholes” in the Web-based ticket-buying system. For example, suppose one physician’s tickets for today were sold out online, the ticket tout could promise an additional ticket as they have reserved extra tickets. If they cancel some of them, new spaces will appear for the patients within several minutes. On the patients’ side, they perceive there to be a high possibility that physicians collaborate with the ticket touts. In extraordinary cases, people may dress up in medical gowns in large hospitals and pretend that they are reputable physicians, soliciting money from those who desperately need health care services in exchange for fictitious advice. These experiences lead to a common distrust toward physicians.

In one sense, physicians must be responsible for patients and improve their quality of health care. From another perspective, patients are expected to understand their physicians [33]. We inferred from some patient comments that the impatience of physicians may contribute to the overwhelming number of patients and called for mutual understanding between patients and physicians. Though Web-based negative comments will not disappear immediately [34], physicians may not worry too much about them as many patients make comments on impulse when feeling frustrated by their experience [35]. Instead, complaints could be viewed as free advice for both the hospital and physician to enhance the quality of health care provision [36,37]. Thanks to the anonymity and convenience of expressing dissatisfaction online, patients can evaluate their physicians more precisely without too much consideration being given to social context, such as obeying complex social and cultural norms [36]. From this perspective, physicians could be more open to negative comments and learn from their own failures in health care delivery to make further improvement.

### Limitations

This study is not without flaws. First, data were only captured from the locality of Beijing on the Good Doctor website. It is possible that this data only reflects the dissatisfaction for specific online users, and the conclusions may not apply to other small- and medium-sized cities or hospitals. Meanwhile, as medical rating websites have their own bespoke functionality, comments collected may suggest different outcomes. Second, because of the complexity of the Chinese language, where words have dual meanings, our analysis framework may omit some potential attitudes and complaints. Third, it is assumed that negative comments relate to a patient’s real experience. However, it is possible that negative comments are manipulated by competitors. Future research could consider a further qualitative approach; for example, focus group or in-depth interview methods could be used with patients who have rated their physician negatively online. Additionally, the mechanism of how online negative comments affect patient decision making requires more attention from researchers [28,38].

### Conclusions

This study is different from those that have focused on all Web-based comments for hospitals or physicians [39]; in contrast, we strived to explore the factors associated with patient dissatisfaction through rigorous content analysis of negative patient comments.

Due to increasing medical specialization, patients are in a comparatively disadvantaged position compared with physicians. Research on patient vulnerability through factors associated with dissatisfaction is crucial to the quality in delivery of health care services and patient safety. Despite the prevalence of on-site complaints in medical institutions, few patients adopt this approach to voice their complaints. To analyze the factors associated with dissatisfaction, we collected self-reported patient experiences toward certain physicians on the Chinese medical platform, the Good Doctor website. Though comments are expected to be centered on the physician, patients also discussed their overall experience, covering a wide range of issues, including hospital registration and the attitude of nurses or other staff members, to help other patients choose the right physician, hospital, and medical treatment.

Finally, we conducted content analysis to explore the negative comments of patients and found that all patients have individual and unique concerns. Negative factors are identified in almost all stages of the medical consultation. The factors are often connected but also distinct from one other. In addition, individual variations make these factors more complex. Among them, the key complaints received are limited medical consultation time and impatience of physicians. Other complaints include the level of therapeutic effect experienced, poor treatment schemes, incorrect information being provided, and disrespect toward patients. This is somewhat consistent with previous studies on the analysis of Web-based and offline complaints toward physicians [18,40], but with more detail concerning the workflow of health care delivery. Meanwhile, it should be noted that physician behavior is shaped greatly by the national health care allocation and health care system [41]; no simple solution for the improvement of patient satisfaction or sustainable behavior adjustment exists—this requires fundamental change in the Chinese health care system.

## Abbreviations

GP: general practitioner
PRW: physician rating websites
STD: sexually transmitted diseases
